# Measles vaccination in lung transplant candidates

**DOI:** 10.3389/fimmu.2025.1481206

**Published:** 2025-02-19

**Authors:** Мikhail P. Kostinov, Valentina B. Polishchuk, Аleksey А. Ryzhov, Pavel I. Zhuravlev, Natalia A. Karchevskaya, Evgeniy A. Tarabrin, Irina L. Solovieva, Alexander P. Cherdantsev, Izabella A. Khrapunova, Elena P. Foshina

**Affiliations:** ^1^ Laboratory for Vaccination and Immunotherapy of Allergic Diseases, I. I. Mechnikov Research Institute of Vaccines and Sera, Moscow, Russia; ^2^ Department of Epidemiology and Modern Vaccination Technology, I. M. Sechenov First Moscow State Medical University, Moscow, Russia; ^3^ Department of Thoracic Surgery, N. V. Sklifosovsky Research Institute for Emergency Medicine, Moscow, Russia; ^4^ Department of Hospital Surgery, I. M. Sechenov First Moscow State Medical University, Moscow, Russia; ^5^ Department of Pediatrics, Ulyanovsk State University, Ulyanovsk, Russia; ^6^ Laboratory of Immunological Research Methods, I. I. Mechnikov Research Institute of Vaccines and Sera, Moscow, Russia

**Keywords:** bronchopulmonary disorders, lung transplant, measles, vaccination, vaccine induced measles immunity

## Abstract

**Background:**

The incidence of measles is now increasing. Measles is especially dangerous for high-risk individuals, including lung transplant candidates with severe progressive bronchopulmonary disorders.

**Objective:**

The objective of this study was to investigate how vaccine-induced immunity is developed in lung transplant candidates seronegative for measles. In order to study vaccine-induced measles immunity, the study subjects were divided in two groups. The main group consisted of 22 patients (11 males and 11 females) with severe bronchopulmonary disorders, aged 19 to 58. The control group was made up of healthcare providers who were matched with respect to age and gender to the patients in the main group. All study subjects were given a single dose of measles vaccine. Levels of anti-measles IgG antibodies (Ab) were measured by enzyme-linked immunosorbent assay (ELISA) using the VectoMeasles-IgG kit (Russia).

**Results:**

One month after vaccination, both study groups showed a statistically significant increase in anti-measles IgG Ab compared to baseline levels. In the main group, vaccine-induced Ab levels were significantly lower than in the control group (0.41 [0.098; 1.75] IU/mL *vs*. 1.94 [0.96; 3.3] IU/mL; р<0.0001). The rates of seroconversion were 73% and 100% in the main and control groups, respectively. The majority of non-responders (83%) in the main group had restrictive pulmonary disease. One year after vaccination, anti-measles Ab were detected in 36% (5/14) of the patients in the main group.

**Conclusion:**

Administration of a single dose of measles vaccine to seronegative lung transplant candidates with severe progressive bronchopulmonary disorders was safe and resulted in protective levels of antibodies in 73% of patients. One year after vaccination, anti-measles Ab were detected in 36% of the patients, which suggested that a single dose failed to induce a robust immune response in this patient population.

## 1 Introduction

For patients with severe progressive bronchopulmonary disorders who are waiting for organ transplantation, prevention of vaccine-preventable infections (VPI) is of particular importance since they are at high risk of infection-related complications. Alterations in both innate and adaptive immunity lead to an ineffective elimination of microorganisms from the respiratory tract (1, 2). Infections tend to be severe in such patients, they trigger a progression of the underlying disease and increase the risk of death prior to transplant. In the post-transplant period, predisposing factors for severe infections are immunosuppressive therapy given to prevent graft rejection, impaired mucociliary clearance, denervation of the allograft, and suppression of cough reflex (3). A study conducted by L.N. Walti et al. showed that lung transplant recipients are at increased risk for VPI compared to recipients of other solid organs (4). Thus, this is a specific group of patients who require, both before and after organ transplantation, thorough screening for protective immunity against preventable infections (5–10).

Measles, whose incidence is increasing worldwide, is one of such infections. According to the World Health Organization (WHO), in 2023 the WHO European Region experienced a 30-fold rise in measles cases compared with the previous year (11). In immunocompromised patients, measles infection evolves as a severe disease and leads to serious complications in about 80% of cases (12).

In patients with severe progressive bronchopulmonary disorders, measles vaccines can only be administered before organ transplantation since vaccination with live attenuated vaccines is not recommended in recipients of solid organs (6–8). The exception to this is non-immunized pediatric recipients of kidney or liver transplants who are receiving very low doses of immunosuppressive agents and have no signs of graft rejection (13, 14).

Our literature search did not identify any reports concerning the development and duration of measles immunity in patients with severe bronchopulmonary disorders. Moreover, new focal outbreaks of measles support the need to investigate this issue and to justify the necessity of vaccination for patients with various bronchopulmonary disorders.

The objective of the study was to examine the specific features of vaccine-induced measles immunity in lung transplant candidates with severe progressive bronchopulmonary disorders.

## 2 Materials and methods

### 2.1 Patients

The study included 44 participants who were divided into two groups.

The main group consisted of 22 patients (11 males and 11 females), aged 19 to 58 (median [Me] age 32 [28; 38] years old), with severe progressive bronchopulmonary disorders who were candidates for lung transplant and were negative for anti-measles IgG antibodies (IgG Ab). Overall, 27% (6/22) of the patients had obstructive pulmonary disease, 5% (1/22) had vascular pulmonary disease, 32% (7/22) had cystic fibrosis, and 36% (8/22) suffered from restrictive pulmonary disease. The patients with obstructive pulmonary disease and cystic fibrosis received bronchodilators and mucolytic agents. The patients with cystic fibrosis were additionally treated with antibiotics, depending on pathogen susceptibility. The patients with idiopathic pulmonary fibrosis received antifibrotic agents (pirfenidone or nintedanib), and the patients with fibrosis caused by extrinsic allergic alveolitis constantly received small doses of systemic corticosteroids (10 mg per day).

The control group of seronegative individuals was made up following measles immunity testing in an organized community. These subjects had no contraindications to measles vaccination; they had been vaccinated in their childhood in accordance with the Russian national standard immunization schedule. The control group was comprised of 22 healthcare providers seronegative for measles (11 males and 11 females), aged 21 to 60 (Me 32 [28; 38]) who were matched with respect to age and gender to the patients in the main group.

Inclusion criteria: The main group was comprised of adult patients with severe bronchopulmonary disorders who were seronegative for measles and, based on their screening results, were placed on the lung transplant waiting list. All the patients gave voluntary consent to participate in the study and signed a consent form. The control group consisted of healthy healthcare providers who were seronegative for measles and signed a consent form to provide their voluntary consent to receive vaccination. Non-inclusion criteria: Refusal to participate in the study.

The study was conducted in compliance with the guidelines of the Declaration of Helsinki. The study protocol was approved by the Local Ethics Council at the Federal State Budgetary Scientific Institution I.I. Mechnikov Research Institute of Vaccines and Sera (protocol # 1 dated April 21, 2020). All the study subjects provided informed consent to participate in the study.

### 2.2 Methods

#### 2.2.1 Collection of biological samples

Blood samples for IgG Ab were obtained before and 1 and 12 months after vaccination in the main group and before and 1 month after vaccination in the control group. Biological samples were processed by the Laboratory for Vaccination and Immunotherapy of Allergic Diseases at the Federal State Budgetary Scientific Institution I.I. Mechnikov Research Institute of Vaccines and Sera using the scientific equipment of the Collective Usage Center of the institute.

#### 2.2.2 Determination of IgG Ab

Serum levels of anti-measles IgG Ab were measured by enzyme-linked immunosorbent assay (ELISA) using the VectoMeasles-IgG kit (Russia). According to the guidelines for assay of IgG Ab, serum levels of anti-measles IgG Ab below 0.12 IU/mL were considered negative. Equivocal results (levels of IgG Ab ranging between 0.12 and 0.17 IU/mL) were also considered negative.

#### 2.2.3 Statistical analysis

Descriptive statistics for quantitative variables included the median and interquartile range, while descriptive statistics for qualitative variables included proportions and their 95% confidence intervals estimated using the Clopper-Pearson method and numbers of subjects with the specified characteristics (n) in the study group (N). The Wilcoxon and the McNemar’s tests were used to investigate the differences between two related samples in terms of quantitative and qualitative variables, respectively. The comparison of quantitative variables was carried out using the Mann-Whitney test for two independent samples and the Kruskal-Wallis test for three independent samples. Qualitative variables in two independent samples were compared using the exact Fisher’s test. All calculations were done using open source statistical programming environment R (Project R for statistical computing) (v.4.0.4).

## 3 Results

### 3.1 Levels of vaccine-induced anti-measles IgG Ab in the study groups

In lung transplant candidates with severe bronchopulmonary disorders, measles vaccination was not associated with any adverse reactions either within the first 7-10 days or within 25-30 days after vaccine administration. Similarly, no unusual reactions were reported after vaccine administration in the control group.

No cases of measles were observed during the study period.

**Figure 1:**
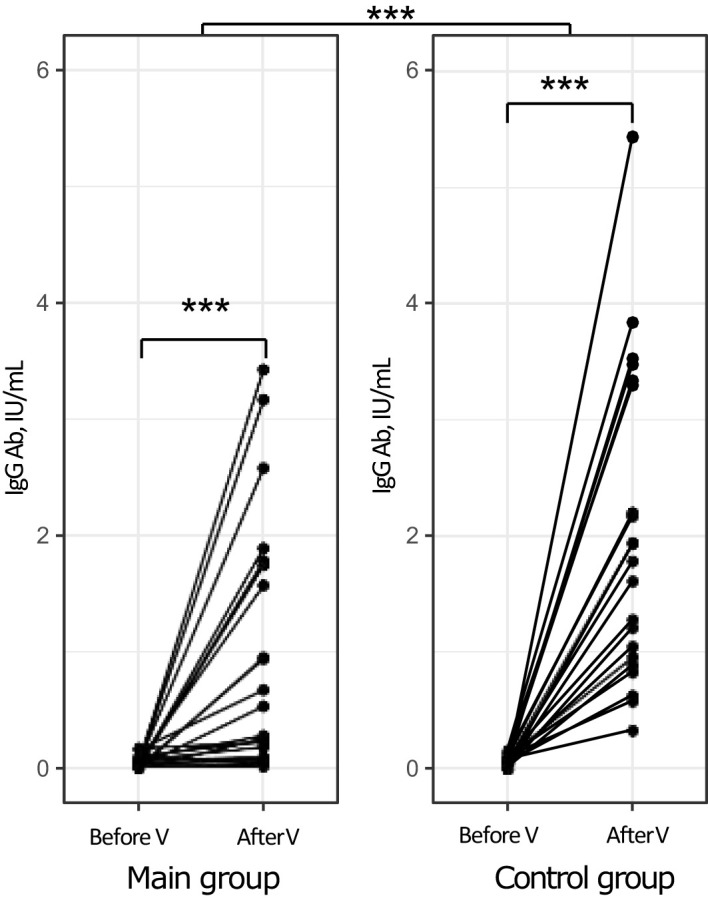
Levels of anti-measles IgG Ab in the study groups before and after vaccination (individual values). ***p<0.001, V, vaccination.

The baseline levels of anti-measles IgG Ab were similar in the main and control groups (р=0.27) (**Figure 1**). One month after vaccination, there was a statistically significant rise in anti-measles IgG Ab levels compared to baseline both in the group of patients with severe bronchopulmonary disorders and in the control group: from 0.05 (0.015; 0.078) IU/mL to 0.41 (0.098; 1.75) IU/mL (р<0.001) in the main group and from 0.07 (0.04; 0.09) IU/mL to 1.94 (0.96; 3.3) IU/mL in the control group (р<0.0001). There was also a statistically significant difference in vaccine-induced IgG Ab levels between the study groups (р<0.0001).

One month following measles vaccination, the proportion of patients who had achieved protective levels of anti-measles IgG Ab (≥0.18 IU/mL) was 73 [50-89]% (16/22) (р<0.001 compared to baseline, as assessed using the McNemar’s test). In contrast, all subjects in the control group (100 [85-100]%) had protective levels of anti-measles IgG Ab (р<0.001 compared to baseline, as assessed using the McNemar’s test) (р=0.021 for between-group comparison).

In the main group, the proportions of male and female patients with protective levels of anti-measles IgG Ab one month after vaccination were 63.6% (7/11) and 81.8% (9/11), respectively (р=0.64).

The between-group comparison revealed that the mean level of vaccine-induced anti-measles IgG Ab in male subjects was higher in the control group compared to that in the main group: 1.94 (0.63; 3,48) IU/mL *vs*. 0.28 (0.09; 0.94) IU/mL, respectively (р=0.008). In female subjects, the mean levels of vaccine-induced anti-measles IgG Ab were 0.965 (0.19; 1.79) IU/mL in the main group and 1.93 (1.28; 3.22) IU/mL in the control group (р=0.053) (**Table 1**).

**Table 1:** Changes over time in levels of anti-measles IgG Ab by gender in the study groups.

Study group	Gender	Level of anti-measles IgG Ab (IU/mL)		
		Before vaccination	At 1 month after vaccination	Comparison over time
Main group	Male	0.046 (0.015; 0.078)	0.284 (0.089; 0.942)	р=0.0044
	Female	0.056 (0.003; 0.102)	0.955 (0.187; 1.786)	р=0.0076
	Intra-group comparison	р=0.84	р=0.45	–
Control group	Male	0.08 (0.05; 0.1)	1.94 (0.63; 3.48)	р=0.0033
	Female	0.05 (0.01; 0.09)	1.93 (1.28; 3.22)	р=0.0033
	Intra-group comparison	р=0.094	р=0.72	–
Inter-group comparison	Male	р=0.053	р=0.008	–
	Female	р=0.87	р=0.053	–

### 3.2 Levels of vaccine-induced anti-measles IgG Ab by type of disease in the main group

Six lung transplant candidates participating in the study had obstructive pulmonary disease, seven patients had cystic fibrosis, eight patients had restrictive pulmonary disease, and one patient suffered from vascular pulmonary disease. One month following vaccination, the patient with vascular pulmonary disease had a protective level of anti-measles IgG Ab (3.2 IU/mL). **Table 2** shows the proportions of responders among patients with obstructive pulmonary disease, restrictive pulmonary disease, and cystic fibrosis. One month post-vaccination, the proportions of seropositive patients were 100% and 86% among individuals with obstructive pulmonary disease and cystic fibrosis, respectively. In the subgroup of patients with restrictive pulmonary disease, the proportion of patients whose antibody levels were over the threshold value was only 38%, which was significantly different from those in other groups. Of note, patients with cystic fibrosis were younger than patients with obstructive or restrictive pulmonary disease.

**Table 2:** Proportion (%) of seropositive patients one month after measles vaccination by type of disease.

Study group	Age, years	Proportion of patients with protective levels of IgG Ab (>0.18 IU/mL)*	
		At month 1 after vaccination	Comparison to baseline
^1^Obstructive (n=6)	41 (32; 44)	100 [54-100]	р=0.04
^2^ Cystic fibrosis (n=7)	27 (24; 29)	86 [42-100]	р=0.04
^3^ Restrictive (n=8)	34 (32; 39)	38 [9-76]	р=0.25
Inter-group comparison	р^1-2^ = 0.018 р^1-3^ = 0.36 р^2-3^ = 0.004	р^1-2^ = 1.0 р^1-3^ = 0.031 р^2-3^ = 0.12	–

* The proportions of subjects with the specified characteristic and their 95% confidence intervals estimated using the Clopper-Pearson method.

**Figure 2:**
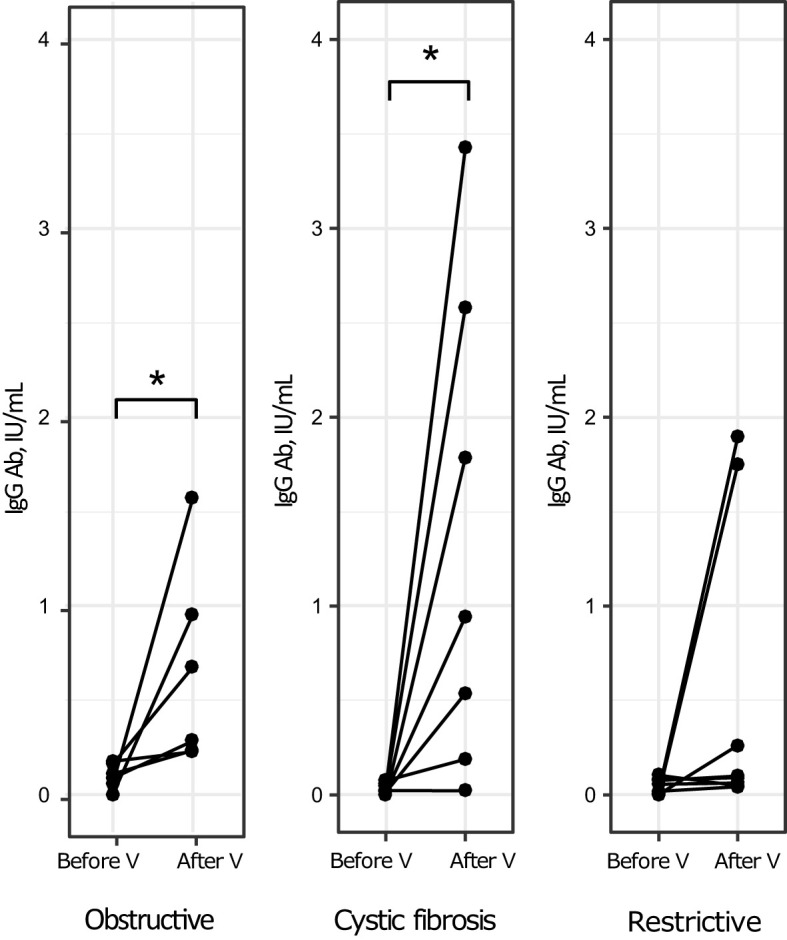
Levels of anti-measles IgG Ab before and after vaccination by type of disease (individual values). *p<0.05, V, vaccination.

In the subgroups of patients with obstructive pulmonary disease and cystic fibrosis there were similar trends in anti-measles IgG Ab response, i.e. a statistically significant increase compared to baseline, which is shown in **Figure 2**. Patients with restrictive pulmonary disease also showed an increase in anti-measles IgG Ab, but no statistically significant difference compared to baseline was observed (p=0.07).

Patients with obstructive pulmonary disease, restrictive pulmonary disease, and cystic fibrosis had similar levels of vaccine-induced anti-measles IgG Ab (H (2, N=21) =2.68, p = 0.26).

Compared to healthy control subjects, patients with obstructive pulmonary disease and restrictive pulmonary disease had significantly lower vaccine-induced anti-measles Ab levels (**Table 3**). Patients with cystic fibrosis and healthy subjects had similar levels of vaccine-induced IgG Ab.

**Table 3:** Levels of vaccine-induced anti-measles IgG Ab in the main group (by type of disease) and in the control group.

Main group		Control group	Intra-group comparison over time
Disease	Level of vaccine-induced IgG Ab (IU/mL)	Level of vaccine-induced IgG Ab (IU/mL)	
Obstructive (n=6)	0.48 (0.23; 0.96)	1.94 (0.96; 3.3)	р=0.005
Cystic fibrosis (n=7)	0.94 (0.19; 2.58)		р=0.154
Restrictive (n=8)	0.09 (0.05; 1.00)		р=0.0012

Of note, 67% (4/6) of the lung transplant candidates who remained seronegative for measles one month after vaccination were males. Patients with positive and negative antibody response to vaccination were similar in age: 30.5 (27.5; 35,5) years old *vs*. 36 (33; 39) years old, respectively (р=0.238). Most of the patients with a negative antibody response to vaccination (83%; 5/6) had restrictive pulmonary disease and constantly received low doses (10 mg/day) of glucocorticoids (GC). Most of the responders (81%; 13/16) did not receive GC. **Table 4** shows the proportions of patients with protective levels of antibodies among those who received and did not receive GC. However, there was no difference in the level of anti-measles IgG-AT 1 month after vaccination between patients who received and did not receive GCS (p = 0.076).

**Table 4:** Levels of anti-measles IgG Ab and proportions of seropositive patients in the main group at month 1 after vaccination by GC therapy status.

Patient subgroup	IgG Ab level (IU/mL) after vaccination	Proportion (%) of seropositive patients after vaccination
Did not receive GC (n=14)	0.81 (0.23; 1.79)*	93% (13/14)
Received GC (n=8)	0.094 (0.05; 1.00)	38% (3/8)
p level	р=0.076	р=0.011

*p < 0.0012 compared with baseline.

Because of the restrictions imposed by the COVID-19 pandemic, 12 months after measles vaccination we could follow up only 14 lung transplant candidates with severe bronchopulmonary disorders. One month after vaccination, anti-measles Ig Ab were detected in 64.3% of these 14 patients (9/14), while 12 months after vaccine administration only 35.7% (5/14) of the patients remained seropositive (р=0.26, the Fisher’s test). There was also a decline in anti-measles Ab over time: from 0.25 (0.06; 0.68) IU/mL one month post-vaccination to 0.12 (0.03; 0.22) IU/mL 12 months post-vaccination (p=0.4).

During this follow-up period, two patients had lung transplant surgery (5 and 9.5 months post-vaccination). One month after vaccination, both patients had protective levels of Ab and 12 months after vaccine administration only one of them remained seropositive but had a very low level of IgG Ab (0.22 IU/mL).

## 4 Discussion

In Russia, mass vaccination of the population with a single dose of measles vaccine was started in 1968, and revaccination has been carried out since 1987. Currently, according to the national immunization schedule of the Russian Federation, measles vaccination is carried out for children aged 12 months with revaccination at 6 years. Immunization is recommended for all people under 35 years, who have not suffered from measles, have not been vaccinated at all or have been vaccinated once and who have no information on previous measles vaccinations. Immunization is also recommended for adults aged 36-55 if they are at risk.

The increased incidence of measles has led to multiple studies that thoroughly investigated immunity against measles and demonstrated decline in long-term vaccine-induced immunity, especially in young adults (15–21). These reports suggested that two-dose measles vaccination in childhood fails to maintain protective levels of IgG Ab over a long period of time, leading to a discussion on whether a third dose of vaccine is required (22).

In patients with severe bronchopulmonary disorders, the prevalence of individuals seronegative to measles virus in young age groups was also revealed (23, 24).

Our study demonstrated that administration of a single dose of measles vaccine to healthy adults resulted in protective levels of anti-measles IgG Ab in all the subjects. Similar results were reported by other authors (25, 26). A study conducted by A.P. Toptygina et al. showed that measles vaccination of seronegative adults was effective both in non-immunized individuals and in those who had been vaccinated in childhood but failed to maintain protective Ab levels (26). I. Medeni et al. reported slightly different results, showing that the rate of seroconversion after single-dose vaccination against measles ranged from 72.7% to 89.5%, which depended on the amount of previous vaccination (27).

In our study population of patients with severe progressive pulmonary disorders, the proportion of responders was 73%, which was significantly different from that in the control group (100%; р=0.021). This is to be expected, as chronic diseases are accompanied by disturbances in molecular and cellular mechanisms, which can affect the immune response in the post-vaccination period as well (28–31). One month after vaccination, there was a significant elevation in anti-measles IgG Ab levels in both groups. However, patients with bronchopulmonary disorders had significantly lower Ab levels than healthy controls (р<0.0001). Moreover, male patients had lower levels of vaccine-induced antibodies than heathy males (р=0.008). It should be emphasized that the previously identified gender differences in lower levels of measles antibodies in men suggest that concomitant chronic pathology further aggravates the formation of adaptive immunity (32).

After the participants in the main group were stratified by type of disease, the subgroups of patients with obstructive pulmonary disease, patients with cystic fibrosis and healthy controls had similar proportions of individuals who were seropositive for measles one month post-vaccination. Although it should be noted that the values of post-vaccination antibodies in the indicated groups of patients were lower than in healthy people and this may be a signal that they may lose their protective properties in the near future. In the subgroup of patients with restrictive pulmonary disease, the rate of seroconversion was only 38%, which was significantly different from those in the control group and in the subgroups of patients with other pulmonary disorders. It is probably explained by treatment given to these patients, including GC.

We did not identify any reports about the development of vaccine-induced measles immunity in patients with bronchopulmonary disorders. The analysis of papers investigating the immunological effectiveness of measles vaccinations within the first months after immunization in other groups of immunocompromised patients produced inconsistent findings. Some studies showed that in seronegative patients who had undergone hematopoietic stem-cell transplantation (HSCT) administration of a single dose of measles vaccine resulted in a positive antibody response in 64-69% of the cases (33, 34). In one of these studies, administration of a second dose of measles vaccine to individuals who remained seronegative after the first vaccination resulted in the production of measles IgG Ab at a protective level in all patients (34). At the same time, T. Aoki et al. reported that only 19% of HSCT recipients had protective anti-measles Ab after receiving two doses of vaccine administered one month apart (35). Another study demonstrated an 81% seroconversion rate in a group of HIV patients three months after vaccination, which was similar to the rate reported in the control group (86%) (36).

Our study showed that most patients failed to maintain protective levels of anti-measles antibodies in the long-term period after vaccination, when the proportion of patients with protective antibody levels was 36%. A rapid loss of IgG Ab in immunocompromised patients was also reported in some other studies. Thus, in a group of HIV patients, the proportion of seropositive individuals dropped from 81% at month 3 after vaccination to 34% at year 1 post-vaccination (36). For HSCT recipients who were seropositive before allogeneic transplantation, the probability of maintaining protective levels of anti-measles IgG Ab two years after surgery was 60.6% (32).

Nowadays, with measles cases being reported in all countries, monitoring anti-measles Ab over time should be a mandatory procedure for all lung transplant candidates. After vaccination, IgG Ab levels should be measured not only at month 1, but also at months 3-6 in order to identify individuals who have lost IgG Ab and to give them a booster dose of measles vaccine in a timely manner. Special attention should be given to patients with restrictive pulmonary disease receiving GC.

Prospects for continuation of the study based on our results and analysis of literature sources allow us to conclude that in this cohort of patients with severe bronchopulmonary pathology it is more appropriate to administer two doses of measles vaccine, which will promote intensive production of antibodies and their long-term persistence.

## 5 Conclusion

Our study showed that administration of a single dose of measles vaccine to seronegative lung transplant candidates with severe progressive bronchopulmonary disorders was safe and resulted in protective levels of antibodies in 73% of patients. Twelve months after vaccination, the proportion of patients with undetectable levels of anti-measles IgG Ab rose to 64%, which suggested that a single dose failed to induce a robust immune response in this patient population.

## Data Availability

The raw data supporting the conclusions of this article will be made available by the authors, without undue reservation.
